# Digital phenotyping captures autism-associated behaviors in preschool- and school-age autistic children with and without co-occurring ADHD

**DOI:** 10.1038/s41598-026-65395-7

**Published:** 2026-08-12

**Authors:** Vikram Aikat, Kimberly L. H. Carpenter, Juan Matias Di Martino, Pradeep Raj Krishnappa Babu, Steven Espinosa, Naomi Davis, Lauren Franz, Marina Spanos, Guillermo Sapiro, Geraldine Dawson

**Affiliations:** 1 Department of Computer Science, Duke University, 140 Science Dr., Durham, NC 27708 USA; 2 Department of Psychiatry and Behavioral Sciences, Duke University, Durham, NC USA; 3 Duke Center for Autism and Brain Development, Duke University, Durham, NC USA; 4 Department of Informatics and Artificial Intelligence, Universidad Católica del Uruguay, Montevideo, Uruguay; 5 Office of Information Technology, Duke University, Durham, NC USA; 6 Duke Global Health Institute, Duke University, Durham, NC USA; 7 Department of Electrical and Computer Engineering, Princeton University, Princeton, NJ USA

**Keywords:** Autism, Digital phenotyping, Co-occurring conditions, Computer vision, Preschool- and school-age, Autism and co-occurring ADHD, Diseases, Neuroscience, Psychology, Psychology

## Abstract

There is a need for scalable, objective assessment tools to quantify autism-related behaviors in preschool- and school-age children. A significant challenge is the heterogeneous presentation of autism, driven in part by co-occurring conditions such as Attention-Deficit/Hyperactivity Disorder (ADHD). Tools intended for autism must therefore be tested in samples that include ADHD and other comorbidities, not only in autism-versus-neurotypical comparisons. SenseToKnow, a digital phenotyping app, quantifies autism-related behaviors using computer vision, tactile sensors, and machine learning, distinguishing autistic and neurotypical toddlers. We administered SenseToKnow to 183 children aged 40–100 months (3.3–8.3 years): 41 neurotypical, 48 ADHD, 53 autism, and 41 co-occurring autism and ADHD. Two complementary analyses converged. In age-adjusted group comparisons, autistic children, with and without ADHD, exhibited different SenseToKnow features compared to neurotypical and ADHD children, while autistic children with and without ADHD did not differ; children with ADHD alone differed from neurotypical children, particularly during nonsocial stimuli. Furthermore, in regression modeling, autism status was associated with 21 of 23 SenseToKnow features and ADHD status with none. Across both analysis, SenseToKnow features tracked autism status rather than ADHD status, even when the two co-occurred. These results demonstrate that SenseToKnow captures autism-associated behaviors even in the presence of co-occurring ADHD.

**Supplementary Information:**

The online version contains supplementary material available at https://doi.org/10.1038/s41598-026-65395-7.

## Introduction

There is a need for objective, quantitative methods to support screening and characterization of autism-related behaviors in preschool- and school-age children^1,2^. The pathway towards a clinical diagnosis of autism frequently extends for years after initial concern, in part due to heterogeneity in autism presentation and co-occurrence with other neurodevelopmental conditions^3–5^. Approximately 40–60% of autistic individuals are also diagnosed with Attention-Deficit/Hyperactivity Disorder (ADHD)^6–10^. Co-occurring ADHD has been linked to delayed autism diagnoses in approximately 20% of children, with average delays of 12–20 months compared to autistic children without ADHD^11,12^. These delays underscore the need for assessment tools capable of detecting autism-related behaviors in the presence of co-occurring conditions, such as ADHD.

Jaiswal et al.^13^ trained machine learning models on caregiver report data from the National Survey of Children’s Health and found strong specificity and sensitivity when distinguishing children with a neurodevelopmental condition (autism or ADHD) from neurotypical children but found relatively low performance when attempting to separate autism, ADHD, co-occurring autism and ADHD, and neurotypical children. This highlights the overlapping nature of caregiver report responses across these diagnostic groups and the need for objective assessment tools to measure behaviors associated with autism in the context of co-occurring conditions.

While both autism and ADHD are classified as neurodevelopmental conditions, with frequent co-occurrence, they each present uniquely, with a mix of overlapping and distinctive phenotypic profiles through behaviors such as attention problems, cognitive development, and social impairment^14,15^. Therefore, an important question is whether digital phenotyping measures capture autism-associated behavioral patterns when comparison samples include other neurodevelopmental conditions such as ADHD, rather than only neurotypical children.

Many autism assessment tools rely on caregiver report, which can be shaped by respondent characteristics such as mental health, education, literacy, and socioeconomic status^16–20^. Objective app-based measures are intended to complement rather than replace caregiver report. In response, recent work has focused on scalable, objective assessment approaches based on speech and language^21,22^, computer vision analysis (CVA)^23,24^, and eye tracking^25,26^. SenseToKnow^27^, a mobile app that requires only the front-facing camera of a mobile device or tablet, uses computer vision analysis and touchscreen-based features to capture autism-related behaviors across clinically relevant domains such as social attention, facial expressions, and visual-motor coordination. SenseToKnow has demonstrated high sensitivity and specificity for autism detection in toddlers in both a primary care^27^ and caregiver-administered home settings^28^. Prior SenseToKnow studies have shown that, relative to neurotypical peers, autistic toddlers exhibit reduced response to name^29^, reduced and more diffuse social attention with poorer gaze-following of speech^30^, increased head-movement rate and acceleration^31^, altered head and facial movement complexity^32,33^, lower overall attention to the stimuli^34^, and reduced bubble-popping skill^35^.

These findings were largely replicated in a study that administered SenseToKnow to autistic and neurotypical children aged 3 to 8 years^36^. Recent digital phenotyping work has begun to examine co-occurring ADHD, reporting that autistic-versus-neurotypical differences persisted regardless of co-occurring conditions^37^, but analyzed only autistic participants with and without ADHD rather than including an ADHD-only group. Direct four-group comparisons that place autism, ADHD, their co-occurrence, and neurotypical development in a single model remain rare; related eye-tracking studies that did include an ADHD-only group^38,39^ found social-attention differences as the strongest difference between those with autism and ADHD. The impact of co-occurring neurodevelopmental conditions, particularly on CVA-derived measures, has not been directly quantified. Lord and Wilson^40^, in a commentary on SenseToKnow, raised the specific concern that digital phenotyping tools may detect general neurodevelopmental differences rather than autism-specific behaviors, a concern that can only be addressed by evaluating the app in clinically heterogeneous samples that include non-autistic neurodevelopmental conditions like ADHD. The present study begins to address this open question.

Specifically, we explored whether SenseToKnow features differ between autistic and neurotypical children even when autism co-occurs with ADHD, and whether app features differ between children with ADHD alone from both neurotypical and autistic peers. We applied an interpretable linear regression model to quantify the independent effects of autism and ADHD status on app feature outcomes, and examined associations of app features with adaptive behavior and autism-related symptom severity using the Vineland Adaptive Behavior Scales (VABS-3^41^ and the Social Responsiveness Scale (SRS-2 ^42^). Together, these analyses characterized how SenseToKnow features present in a more heterogeneous group of children and quantified the independent contributions of autism and ADHD status to these digital phenotyping feature outcomes.

## Results

The final sample comprised 183 children: 41 neurotypical, 48 ADHD, 53 autism, and 41 co-occurring autism+ADHD. Groups differed in age (means 67.9, 77.9, 67.7, and 80.0 months for neurotypical, ADHD, autism, and autism+ADHD respectively) and, as expected from the eligibility criteria, in developmental quotient (DQ; Table 1). All primary analyses are adjusted for age; DQ was not included as a covariate due to its strong association with autism in this cohort. Missing data were feature-specific: response-to-name was available for 127 of 183 children and bubble-popping features for 146; all others were complete. Findings were interpreted by emphasizing consistency across the group-difference, regression analyses, and across related features.

### Group differences in SenseToKnow features

**Fig. 1:**
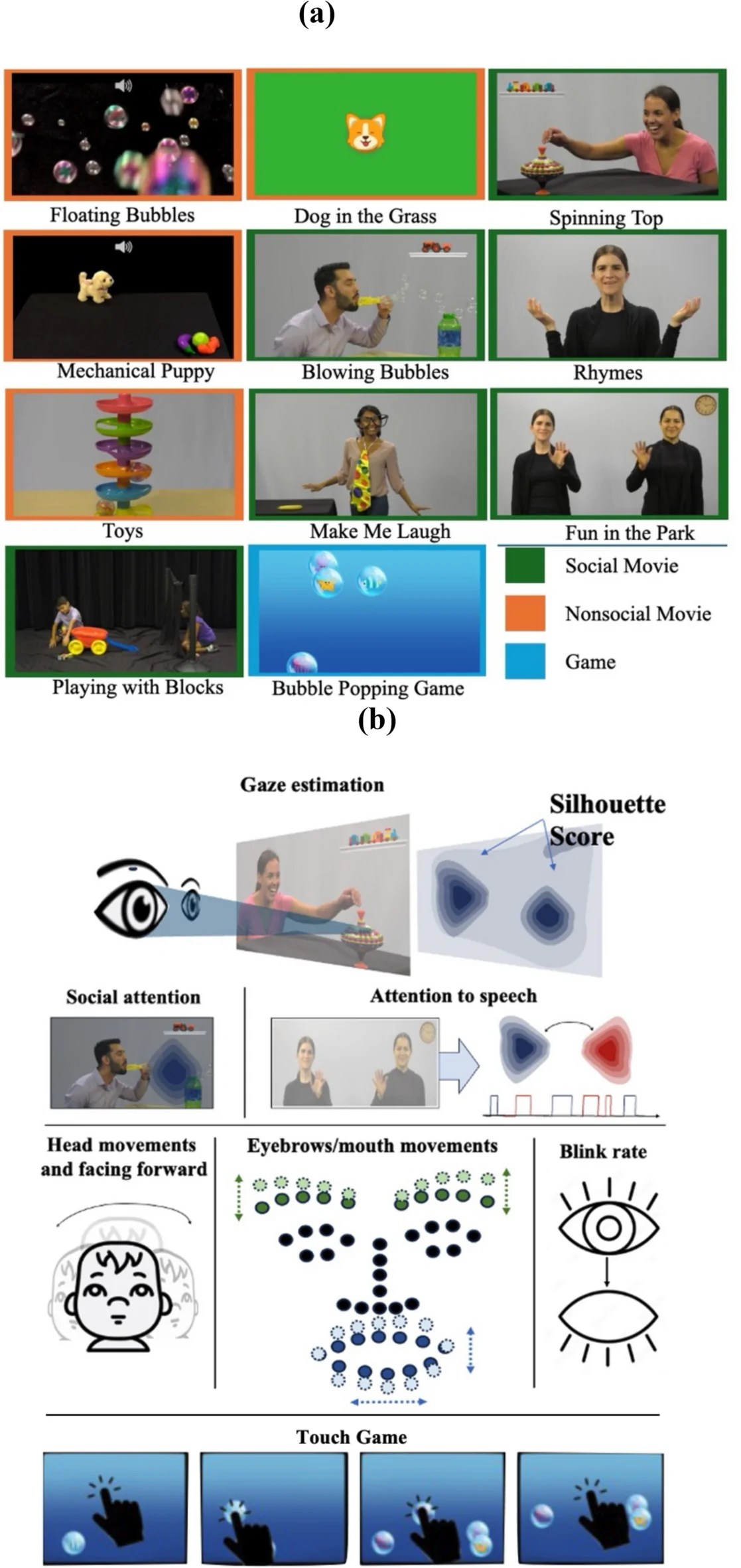
Overview of SenseToKnow app administration. (**a**) Set of movies the participant views on the tablet presented during clinical administration, shown in order (left to right, top to bottom). (**b**) Feature extraction from participant’s video and touch game summarized.

We compared the four diagnostic groups on each of the 23 SenseToKnow features using analysis of covariance with age as a covariate (Table 2). Of all 115 group-level comparisons, 49 were significant before FDR correction and 37 remained significant after correction; we discuss only those below (Fig. 1).

Comparing children with ADHD and autistic children with co-occurring ADHD, the co-occurring group showed reduced correlation between the child’s gaze direction and the speaker’s position (gaze-speech correlation; 0.36 [SD 0.20] versus 0.47 [0.15]; *p* = 0.002), a lower proportion facing forward during social movies (86.5% [14.1] versus 93.5% [9.4]; *p* = 0.006), a higher social head-movement rate (1.74 [1.69] versus 0.73 [0.63]; *p* < 0.001) and acceleration (*p* < 0.001), and lower social head-movement complexity (17.3 [9.2] versus 24.8 [8.1]; *p* < 0.001). These differences survived correction. Conducting similar analyses comparing autistic children with and without co-occurring ADHD, no feature differed between the two groups, and none reached even nominal significance before FDR correction.

**Fig. 2:**
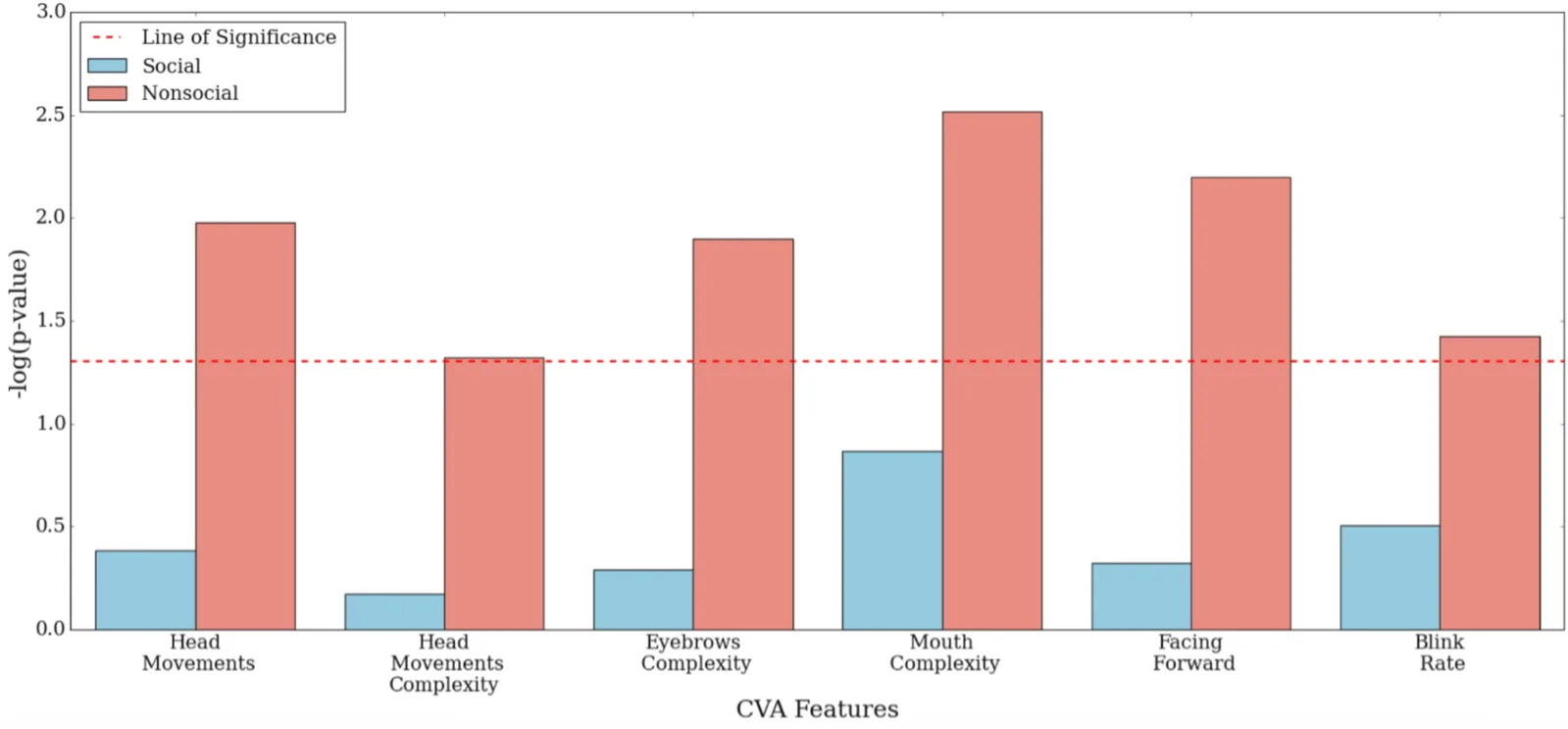
Results of ANCOVA with age as a covariate between ADHD and neurotypical participants during social and nonsocial stimuli across features. -log10 of p-values shown, along with line of significance.

Using the same age-adjusted model, children with ADHD were distinct from neurotypical children primarily during nonsocial movies, a pattern not observed during social movies (Fig. 2). During nonsocial movies, relative to neurotypical children, they exhibited a higher head-movement rate (1.37 [1.69] versus 0.87 [0.73]; *p* = 0.012) and acceleration (*p* = 0.009), reduced mouth complexity (25.4 [5.7] versus 27.8 [3.2]; *p* = 0.003) and eyebrow complexity (26.1 [5.8] versus 28.1 [3.3]; *p* = 0.013), lower proportion facing forward (87.3% [13.7] versus 92.6% [7.3]; *p* = 0.006), reduced gaze silhouette score (0.51 [0.09] versus 0.57 [0.08]; *p* = 0.005), and greater applied touch force (median 0.12 [IQR 0.08 to 0.23] versus 0.09 [0.04 to 0.13]; *p* = 0.009). Differences during social movies were minimal.

Comparisons between autistic and ADHD participants revealed widespread significant group differences. Specifically, autistic children exhibited reduced gaze-speech correlation (0.31 [0.21] versus 0.47 [0.15]; *p* < 0.001), a lower proportion facing forward during social movies (81.9% [17.1] versus 93.5% [9.4]; *p* = 0.002), lower social head-movement complexity (17.1 [9.9] versus 24.8 [8.1]; *p* = 0.002) and nonsocial head-movement complexity (6.8 [4.3] versus 10.8 [5.9]; *p* = 0.003), reduced social eyebrow complexity (26.0 [7.4] versus 30.5 [3.8]; *p* = 0.005), social mouth complexity (24.9 [6.7] versus 29.0 [3.3]; *p* = 0.008), and nonsocial eyebrow complexity (21.5 [7.8] versus 26.1 [5.8]; *p* = 0.008), and a higher social blink rate (*p* = 0.013).

Autistic children with co-occurring ADHD differed from neurotypical children across most domains. Relative to neurotypical children, they showed reduced gaze to social stimuli (0.48 [0.08] versus 0.52 [0.07]; *p* = 0.004), reduced gaze-speech correlation (0.36 [0.20] versus 0.46 [0.16]; *p* = 0.002), and a reduced gaze silhouette score (0.48 [0.10] versus 0.57 [0.08]; *p* < 0.001). They showed a higher head-movement rate during social (1.74 [1.69] versus 0.81 [0.97]; *p* < 0.001) and nonsocial movies (1.85 [1.80] versus 0.87 [0.73]; *p* < 0.001), with corresponding acceleration (both *p* < 0.001), and lower head-movement complexity during social (17.3 [9.2] versus 24.5 [7.4]; *p* < 0.001) and nonsocial movies (8.2 [5.7] versus 11.9 [5.0]; *p* < 0.001). They also faced forward less during social (86.5% [14.1] versus 94.2% [6.9]; *p* = 0.002) and nonsocial movies (85.1% [12.1] versus 92.6% [7.3]; *p* < 0.001), and exerted greater applied touch force (median 0.18 [IQR 0.06 to 0.36] versus 0.09 [0.04 to 0.13]; *p* < 0.001). Reduced nonsocial mouth and eyebrow complexity, a lower popping rate, and greater exploratory touch were also observed (all *p* < 0.05).

### Correlation with age and clinical measures

These correlation analyses were exploratory and hypothesis-generating. Consistent with their exploratory nature, we did not apply formal multiple-comparison correction to all comparisons and instead emphasize the consistency of patterns across related features.

Correlations between app features and age were computed within each diagnostic group and are summarized in Supplementary Table S1. Across groups, response-to-name, gaze, and blink features showed minimal association with age, whereas several touch and movement features exhibited age-related trends. Touch popping rate improved with age in the neurodivergent groups (Spearman rho = 0.32 in autism, 0.37 in co-occurring autism and ADHD, and 0.55 in ADHD) but not in neurotypical children (rho = 0.09), and touchscreen exploratory percentage and touch length decreased with age in the neurotypical and ADHD groups (for example, touch length rho = -0.74 in neurotypical children). During social movies, social head-movement complexity increased with age in the autism group (rho = 0.37), a pattern not seen in the co-occurring group (rho = -0.04).

**Fig. 3:**
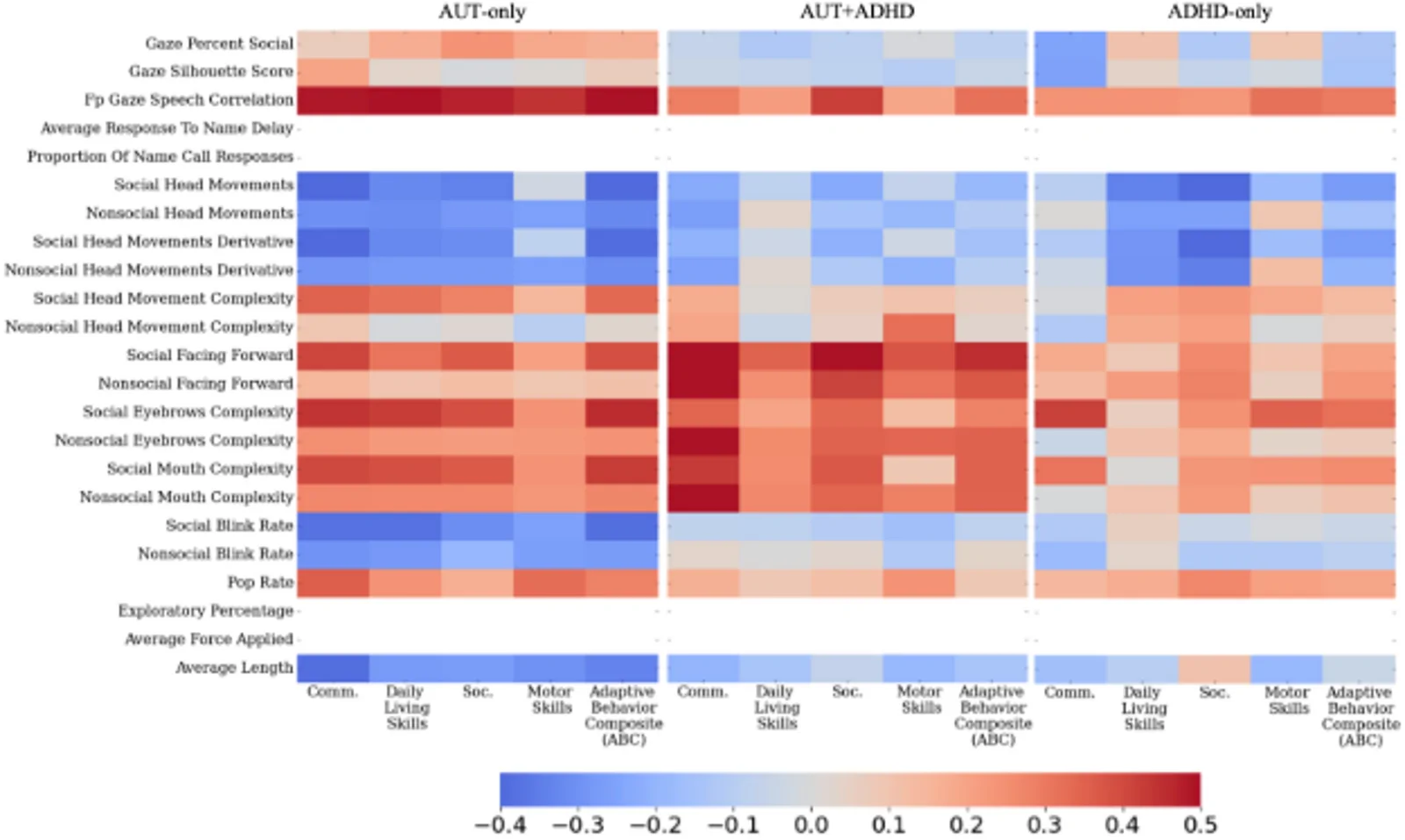
Visualization of Spearman’s correlation between SenseToKnow app features and VABS subdomain scores. Significant positive correlations shown in red and significant negative correlations shown in blue. Correlations that are not significant shown in white.

Associations between adaptive functioning (Vineland-3) and app features, examined within each diagnostic group, are shown in Fig. 3; these analyses were also exploratory. Higher adaptive functioning was generally associated, in the expected direction, with greater gaze-speech correlation, more time facing forward, higher movement complexity, and a higher popping rate, and with lower movement rate, blink rate, and touch length. Directions were largely consistent across groups except for gaze percent social and gaze silhouette score, where effects were small and weakest in the ADHD group. We present these for completeness rather than as evidence of convergent or divergent validity.

We next examined associations between app features and caregiver-reported symptom severity, using the SRS-2 and the ADHD-RS (Table 3). Because these analyses could be interpreted as convergent-validity evidence, we first quantified the overlap between the two scales: across the sample they were correlated at *r* = 0.64 (partial *r* = 0.39 after adjusting for group, age, and developmental quotient), so neither function as a condition-specific criterion, and both are caregiver reports subject to shared response characteristics. Few features were associated with ADHD Rating Scale scores. The strongest associations were reduced gaze silhouette score in neurotypical children (rho = -0.36), higher social blink rate in the autism group (rho = 0.29), and, in the ADHD group during nonsocial movies, reduced gaze-speech correlation (rho = -0.37), lower proportion facing forward (rho = -0.34), and reduced mouth complexity (rho = -0.31). None of these associations survived false-discovery-rate correction.

In contrast, SRS-2 social-awareness scores were associated with multiple app features in the autism groups, and several of these associations survived correction. Within the autism group, children with greater social-awareness severity showed reduced gaze-speech correlation (rho = -0.62), a lower proportion facing forward during social movies (rho = -0.41), lower social head-movement complexity (rho = -0.39) and facial complexity (social eyebrow rho = -0.40, social mouth rho = -0.40), and a higher blink rate (social rho = 0.50, nonsocial rho = 0.41), all surviving correction. Within the co-occurring group, greater severity was associated with a higher head-movement rate and acceleration (social rho = 0.52 and 0.51, nonsocial rho = 0.49 and 0.41), reduced gaze to social stimuli (rho = -0.40) and gaze-speech correlation (rho = -0.39), and a lower proportion facing forward (rho = -0.39), also surviving correction. These associations were unchanged when age was included as a covariate.

Associations with repetitive-behavior severity were fewer. Within the autism group, greater repetitive-behavior severity was associated with reduced gaze-speech correlation (rho = -0.48) and a lower proportion facing forward during social movies (rho = -0.39), both surviving correction, with weaker associations for social head-movement complexity, mouth complexity, and blink rate that did not survive. Within the co-occurring group, no association with repetitive-behavior severity survived correction. Taken together, these patterns suggest that a subset of the app’s social-attention and movement features track autism-related symptom severity, more consistently in the social domain than the repetitive-behavior domain, a possibility for confirmation in larger samples.

### Quantifying the effect of diagnosis on feature outcome through ordinary least squares (OLS) model

Coefficients and significance from the ordinary-least-squares model are presented in Table 4, showing the estimated association of diagnosis and age with each app feature. Autism status was associated with 21 of the 23 features after false-discovery-rate correction, with the exception of response-to-name delay and touchscreen exploratory percentage. Relative to a neurotypical baseline, autism status was associated with reduced gaze metrics (*p* < 0.001), a higher head-movement rate (*p* < 0.001), reduced facial complexity (*p* < 0.001), a lower proportion facing towards the screen (*p* < 0.001), and a higher blink rate (*p* < 0.05). Autism status was also associated with reduced bubble-popping performance, including a lower popping rate, longer average touches, and greater applied force (*p* < 0.05). ADHD status was associated with no feature after correction. Three nominal associations, with gaze silhouette score (*p* = 0.037), popping rate (*p* = 0.025), and applied force (*p* = 0.016), did not survive correction.

**Fig. 4:**
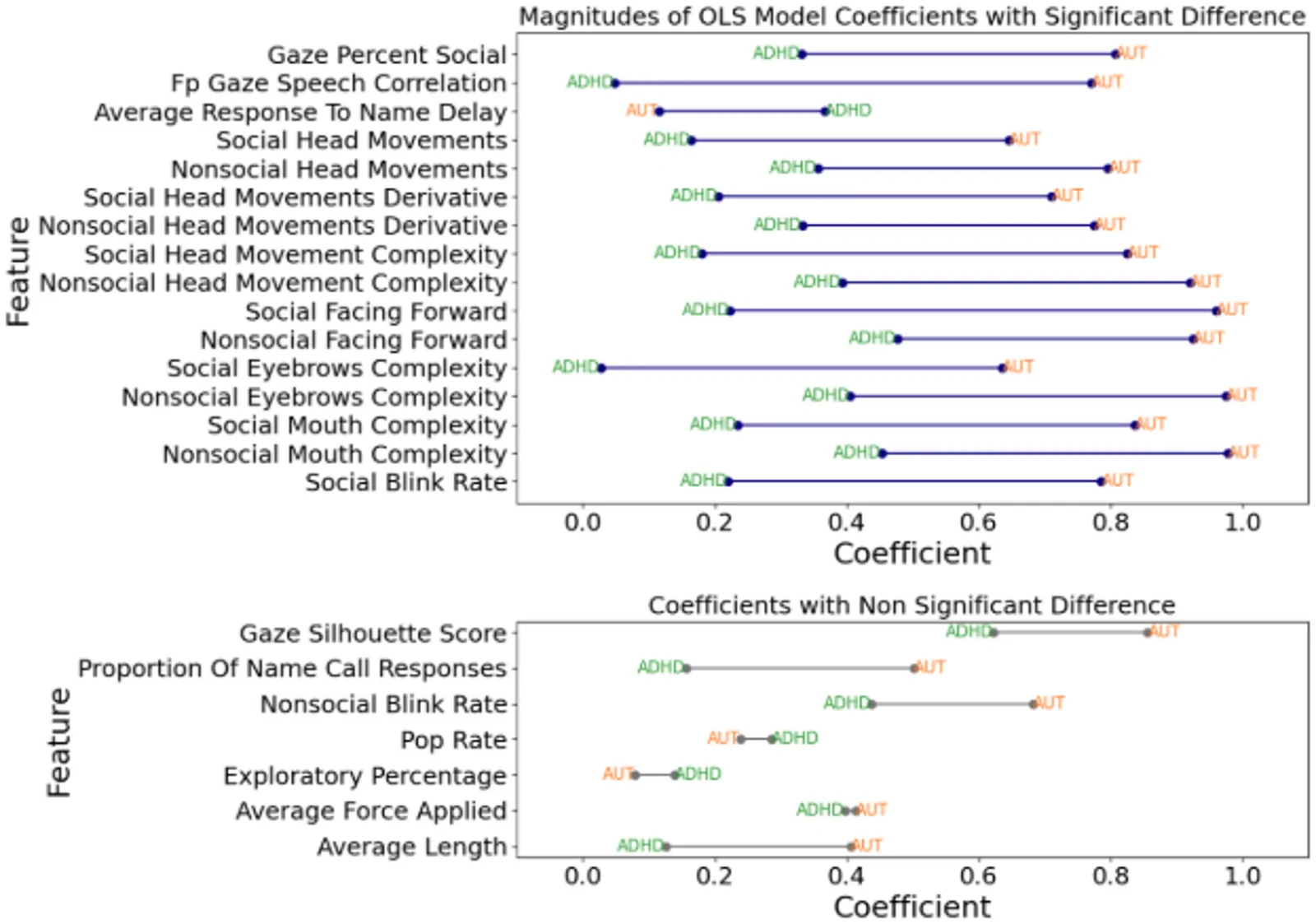
Visualization of difference in OLS coefficient between the effect of having autism and the effect of having ADHD on feature outcome. Lines in blue display significant coefficients.

Age was associated with several features, including gaze-speech correlation, head and facial movement complexity, facing forward, and touch features, but with small coefficients relative to diagnostic status. Comparing the magnitude of the two diagnostic associations descriptively (Fig. 4), autism status had a larger association with feature outcome than ADHD status for nearly all features. Response-to-name latency was the only feature for which the ADHD association exceeded the autism association. We present this as a descriptive comparison of coefficient magnitudes rather than a formal test of specificity.

To assess the effect of co-occurring autism and ADHD, we extended the model to include an autism-by-ADHD interaction term (Table 5). The autism associations were largely unchanged after adding the interaction, and, after correction, no interaction term survived. Seven interactions reached nominal significance, concentrated in nonsocial features including nonsocial head movement, facing forward, and facial complexity, but none survived correction. Adding the co-occurring group also produced nominal ADHD associations with gaze silhouette score, nonsocial mouth complexity, and nonsocial facing forward, none of which survived correction. Age effects remained small and consistent with the earlier analyses.

**Fig. 5:**
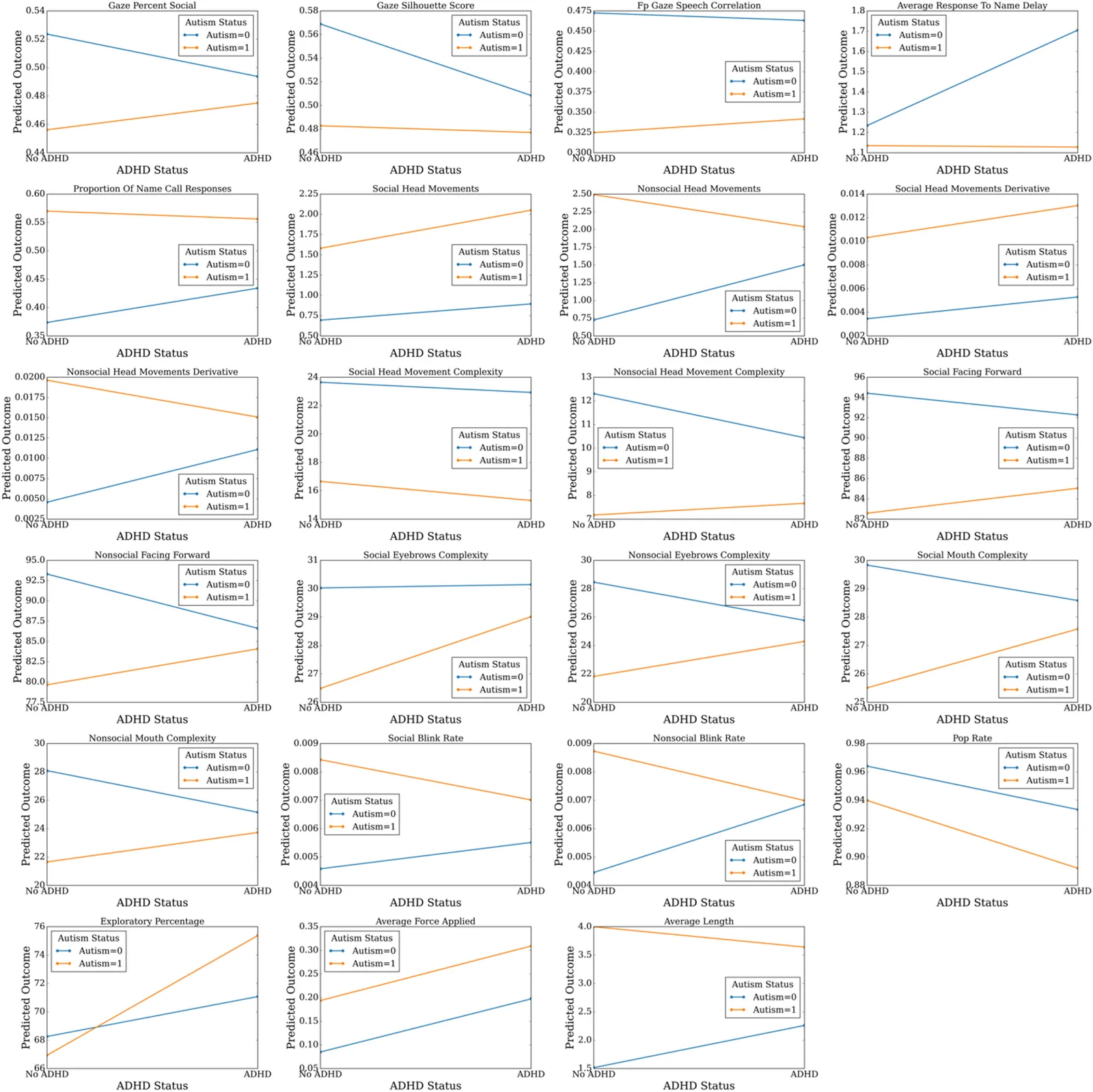
Visualization of the interaction effect between autism and ADHD. The blue line showcases those without autism, and the orange line shows those with autism. The x-axis differentiates ADHD status.

Group-level model-predicted means are visualized in Fig. 5. The co-occurring group most often fell between the autism and ADHD groups, with the exceptions of the social head-movement, popping-rate, and applied-force features, where the co-occurring group was more distinct from neurotypical children than either the autism or ADHD group. We describe these as group-level patterns rather than interaction effects, since no interaction survived correction.

Across both the additive and interaction models, autism status was consistently associated, after correction, with the proportion of name-call responses, gaze features, head-movement features, facial complexity, blink rate, facing forward, and touch length. Together with the absence of any difference between the two autism groups, these analyses indicate that the measured features tracked autism status rather than ADHD status, while remaining agnostic about potential underlying mechanisms associated with co-occurrence.

### Reliability

Ninety-four children completed at least two eligible administrations (10 neurotypical, 27 with ADHD, 34 with autism, and 23 with co-occurring autism and ADHD; median between-visit interval 7.0 days, IQR 2.2 to 14.7). Because the per-group repeat samples were small, reliability was estimated across the pooled repeat sample. Reliability was highest for the movement-complexity and facing-forward features, which were moderate to good (social and nonsocial head movement complexity ICC(A,1) = 0.78 and 0.76; social and nonsocial facing forward 0.74 and 0.71). Gaze-speech correlation, head-movement rate, blink rate, and gaze silhouette score were moderate (ICC 0.50 to 0.67). The weakest features were gaze percent social (0.43), response to name delay (0.28), and the touch features (touch average applied force 0.25, touch average length 0.27). Per-feature ICCs and confidence intervals are provided in Supplementary Table S2.

## Discussion

This study administered SenseToKnow, a digital phenotyping app, with children aged 3–8 years who were diagnosed with autism alone, ADHD alone, or co-occurring autism and ADHD, in comparison with neurotypical children. Prior work has demonstrated the app’s capacity to differentiate autistic toddlers from neurotypical peers^27,28^; the present work examined group differences in SenseToKnow features among autistic children, with and without co-occurring ADHD, versus children with ADHD alone and neurotypical children. Two complementary analyses converged on the same conclusion. Across age-adjusted group comparisons and a regression model including both diagnostic indicators, autism status was more consistently associated with app-feature outcomes than ADHD status. This held even when autism co-occurred with ADHD. Children with and without autism exhibited differences across multiple digital phenotypes after correction for multiple comparisons, whereas group differences involving ADHD alone were fewer and confined to nonsocial features. No feature distinguished autistic children from autistic children with co-occurring ADHD, indicating that the autism-associated digital feature profile was not detectably altered by the presence of co-occurring ADHD, although this also indicates that these features did not capture ADHD-related variation within autism. We further discuss each line of evidence and its implications below. Together, these findings indicate that SenseToKnow features capture autism-associated behaviors in preschool- and school-age children, even when autism and ADHD are co-occurring.

In comparisons between autistic children and children with ADHD alone, robust differences emerged after correction, particularly for features in response to social stimuli. Autistic children showed reduced gaze-speech correlation, a lower proportion facing towards the screen, lower social movement complexity, and a higher social blink rate. Reduced gaze towards social stimuli and gaze-speech correlation were consistent across all comparisons between those with autism and without autism, whereas they did not distinguish children with ADHD alone from neurotypical children. The features that differed between the autism and ADHD groups were behavioral responses to social stimuli and those that differed between children with ADHD and neurotypical children were behavioral responses to nonsocial stimuli. These findings are consistent with eye-tracking results showing reduced social visual attention in autistic individuals with and without ADHD and similar eye-gaze patterns between children with ADHD and neurotypical children^38,39^. Reduced social attention is a core feature of autism^15^. While ADHD is characterized by general attention differences, these results suggest those differences are not specific to social stimuli.

Compared with children with ADHD alone, autistic children showed reduced head and facial movement complexity during social movies, indicating more predictable movement patterns. Prior work has shown that autistic toddlers show atypical facial-movement complexity compared with neurotypical peers^33^; the present study extended this to preschool- and school-age and showed that this complexity differentiated autism from ADHD at the group level, placing it among the autism-associated behaviors rather than a general neurodevelopmental difference. Autistic children with co-occurring ADHD also differed from children with ADHD alone, with a higher head-movement rate and acceleration and lower head-movement complexity, indicating that the reduced head-movement complexity distinguished autism from ADHD whether or not ADHD co-occurred. These movement differences are consistent with evidence that certain motor features may be characteristic of autism rather than a co-occurring impairment^43^, and that movement variability differs in autistic children versus those with ADHD^44^.

During the bubble-popping game, autistic children with co-occurring ADHD popped bubbles at a lower rate than children with ADHD alone, a difference that survived multiple-comparison correction. Descriptively, this group also showed the greatest applied force and the highest exploratory percentage of any group, although the difference in applied force from children with ADHD alone did not survive correction. We describe these as group-level differences rather than an interaction effect, since no autism-by-ADHD interaction survived correction. This aligns with prior reports of touch-feature and motor differences in autistic children with co-occurring ADHD^35^, and with work by Skaletski et al.^45^ and Lin et al.^46^ reporting that fine-motor impairment is most pronounced in children with co-occurring conditions.

Although the SenseToKnow stimuli and computed digital features were designed to elicit and quantify autism-related behaviors, the app also differentiated children with ADHD alone from neurotypical children. Compared with neurotypical children, children with ADHD showed more frequent head movements, reduced facial movement complexity, and reduced attentional engagement (facing forward to the screen) in response to nonsocial stimuli, as well as reduced gaze silhouette score and greater touch force, whereas behavioral differences in response to social stimuli were more limited. This pattern suggests a selectively reduced engagement with nonsocial content in children with ADHD, though further investigation is needed. Prior work has found minimal differences between children with ADHD and neurotypical children in social visual attention and gaze fixation, consistent with our findings^38^, a similarity also reported for social gaze and blink rate in other studies^47,48^.

In the regression analysis, autism status was associated with 21 of the 23 features after correction, whereas ADHD status was associated with none, and no autism-by-ADHD interaction survived correction. This result agrees with the group comparisons, which separated the autism groups from neurotypical and ADHD peers but not from each other. Across both analyses, autism status rather than ADHD status was consistently associated with app feature outcomes. We describe this as a consistent differential association between autism status and the measured behaviors rather than as evidence of a causal account of why the features vary.

We also examined, within each group, how the app features related to symptom severity and adaptive functioning. The SRS-2 and the ADHD Rating Scale overlapped substantially (*r* = 0.64), so neither serves as a condition-specific criterion, and we do not interpret the correlations as establishing convergent or divergent validity. Within this limitation, a coherent pattern was nonetheless visible in the autism groups. Features that separated the diagnostic groups, including gaze-speech correlation, facing forward, social movement complexity, and blink rate, also tracked SRS-2 symptom severity in the expected direction, several of these associations survived correction, and they were robust to age adjustment. Associations with the ADHD Rating Scale were sparser and did not survive correction. This suggests that a subset of the app’s attention and movement features index autism-relevant behavioral variation rather than diagnostic category alone, a possibility for confirmation in larger samples. The substantial overlap between the two caregiver scales is itself informative, since it reflects the documented behavioral overlap between autism and ADHD and cautions against reading either questionnaire as a pure marker of one condition.

This study has several limitations. First, the sample size was modest and both autism and ADHD are behaviorally heterogeneous, so some findings may reflect this particular sample and require replication in larger cohorts. Second, participants were recruited at a single research setting and were predominantly white and male with highly educated caregivers, and all app administrations were in English (although a Spanish version was available), which together limit generalizability to more diverse and multilingual populations. Third, the diagnostic groups differed in age and in cognitive and language level, with the smaller neurotypical group being cognitively advanced relative to the other groups; we adjusted for age in all analyses, but residual confounding by developmental level cannot be excluded, and future studies should recruit larger groups better matched on age and cognitive ability. Fourth, repeatability varied across features. The social-attention and movement-complexity features that carried the autism-associated signal showed moderate to good test-retest reliability, whereas the touch and response-latency features were less reliable and should be interpreted more cautiously. Finally, because autistic children with and without co-occurring ADHD did not exhibit differences in their behavioral features and ADHD-related behavioral differences were confined to responses to nonsocial stimuli, the present findings do not support the use of these features to detect or monitor ADHD. Relatedly, the study was not designed or powered for individual-level diagnostic classification. Whether SenseToKnow features can detect autism, with or without co-occurring ADHD, from those with ADHD or neurotypical development remains an important and open question for prospectively designed studies with larger, independent training and test samples.

In summary, across two analytical approaches, age-adjusted group comparisons and a regression model of both diagnoses, autism status rather than ADHD status was consistently correlated with SenseToKnow feature outcomes, and this held even when autism co-occurred with ADHD. At the group level, autistic children exhibited differences in behavioral features assessed by SenseToKnow from those exhibited by both neurotypical and ADHD peers across social-attention, head movements, facial expressions, and fine-motor domains. Autism diagnostic status had a consistently larger association than ADHD status with the digital phenotypes measured by SenseToKnow. These findings indicate that SenseToKnow digital phenotyping features measure an autism-associated behavioral signal that persists in the presence of co-occurring ADHD.

### Methods

### Participants

Participants were 186 children between 40 and 100 months of age at the time of SenseToKnow app administration (Fig. S1). Children were classified into four groups: neurotypical children (*n* = 41), children diagnosed with autism spectrum disorder alone (“autism”; *n* = 54), children diagnosed with ADHD alone (“ADHD”; *n* = 48), and children diagnosed with co-occurring autism spectrum disorder and ADHD (“autism+ADHD”; *n* = 43). Participants were recruited through community outreach and the research center’s subject registry. At eligibility screening, participants needed to be 36–108 months old. Exclusion criteria included neurological or genetic conditions with known links to autism, such as fragile X, seizure disorders (except for simple febrile seizures or seizure-free status for the prior 12 months), epilepsy, and sensory or motor impairment issues that would interfere with the study administration. Children with a history of neonatal brain damage, or any known environmental circumstances that would account for the presence of autism-related behaviors were excluded as well. Children with conditions with less robust links to autism, such as 16p11.2 CNVs, were not excluded. Children prescribed stimulant medication were required to undergo a 24-hour washout prior to participation.

Additional inclusion criteria for the neurotypical group included average- or above average cognitive ability (IQ > = 70) and average or above average adaptive functioning on the Vineland Adaptive Behavior Scales-3 (VABS-3^41^, as well as no clinical elevations on the (1) Child Behavior Checklist (CBCL^49^— except on the anxiety subscale, (2) ADHD Rating Scale (ADHD-RS^50^, or (3) Social Responsiveness Scale, 2nd Edition (SRS-2^42^). Neurotypical children with a first-degree relative diagnosed with autism or ADHD were excluded, given the heritable nature of these conditions^51,52^.

All autistic participants had a DSM-5 diagnosis of autism spectrum disorder. Prior to March 2020, diagnosis was based on the Autism Diagnostic Interview-Revised (ADI-R^53^ and the Autism Diagnostic Observation Schedule-2 (ADOS-2^54^). During the COVID-19 pandemic, the Brief Observation of Symptoms of Autism (BOSA^55^ was used when the ADOS-2 was not feasible. All diagnoses were made prior to the SenseToKnow administration and played no part in the computation of SenseToKnow features.

Children diagnosed with ADHD met DSM-5 criteria based on clinical evaluation informed by the Mini-International Neuropsychiatric Interview (MINI^56^, and supplemental questions described previously^57–59^. Additional criteria included an IQ *≥* 50, and either total score of < 65 on the SRS-2 or a clinician rule-out of autism.

Valid app data were obtained from all but three participants, one with autism and two with autism+ADHD. Valid data were defined as > 50% of frames with successful facial landmark detection, head pose angles < 35°, and participant distance from device between 60 and 80 cm^28^. The final sample included 183 participants (41 neurotypical, 53 autism, 48 ADHD, 41 autism+ADHD). Demographic characteristics are shown in Table 1. Children in the neurotypical and autism groups were significantly younger than those in the autism+ADHD and ADHD groups (Fig. S1). To assess age-related effects, analyses included age as a covariate.

**Table 1:** Study demographic characteristics.

Demographic variable	Total sample (*N* = 183; 100%)	Neurotypical (*N* = 41; 22.40%)	Autism (*N* = 53; 28.96%)	ADHD (*N* = 48; 26.23%)	Autism +ADHD (*N* = 41; 22.40%)	
Age in months*						
Mean (SD)	73.13 (15.28)	67.77 (15.73)	67.69 (15.70)	77.86 (12.18)	79.97 (12.78)	
DQ*						
Mean (SD)	98.26 (24.42)	116.04 (12.74)	83.00 (22.95)	107.58 (21.82)	89.29 (22.39)	
**Sex**						
Boys (%)	136 (74.32%)	29 (70.73%)	41 (77.36%)	38 (79.17%)	28 (68.29%)	
Girls (%)	47 (25.68%)	12 (29.27%)	12 (22.64%)	10 (20.83%)	13 (31.71%)	
**Race**						
White/Caucasian (%)	136 (74.32%)	35 (85.37%)	32 (60.38%)	33 (68.75%)	36 (87.8%)	
Black or African American (%)	11 (6.01%)	1 (2.44%)	4 (7.55%)	4 (8.33%)	2 (4.88%)	
Asian (%)	9 (4.92%)	1 (2.44%)	6 (11.32%)	1 (2.08%)	1 (2.44%)	
More than one race (%)	22 (12.02%)	3 (7.32%)	8 (15.09%)	9 (18.75%)	2 (4.88%)	
Other (%)	5 (2.73%)	1 (2.44%)	3 (5.66%)	1 (2.08%)	0 (0%)	
**Ethnicity**						
Hispanic\Latino (%)	21 (11.48%)	3 (7.32%)	5 (9.43%)	7 (14.58%)	6 (14.63%)	
Not Hispanic/Latino (%)	162 (88.52%)	38 (92.68%)	48 (90.57%)	41 (85.42%)	35 (85.37%)	
Caregivers’ education level						
High school diploma (%)	4 (2.19%)	1 (2.44%)	2 (3.77%)	0 (0%)	1 (2.44%)	
Some college education (%)	19 (10.38%)	1 (2.44%)	9 (16.98%)	4 (8.33%)	5 (12.2%)	
4-year college degree or more (%)	159 (86.89%)	39 (95.12%)	42 (79.25%)	44 (91.67%)	35 (85.37%)	

* *p* < 0.05 for between-group comparison.

ANOVA and Chi-square tests were used for numerical and categorical comparisons, respectively.

**Table 2:** Group differences using ANCOVA with age as a covariate for each SenseToKnow feature in neurotypical (NT), ADHD, autism, and co-occurring autism and ADHD (autism+ADHD) groups.

Digital phenotype	SenseToKnow feature	ADHD vs. autism +ADHD	NT vs. ADHD	NT vs. autism +ADHD	Autism vs. ADHD	Autism vs. autism +ADHD
		*N* = 89 *p*, $\:{n}_{p}^{2}$	*N* = 89 *p*, $\:{n}_{p}^{2}$	*N* = 82 *p*, $\:{n}_{p}^{2}$	*N* = 101 *p*, $\:{n}_{p}^{2}$	*N* = 94 *p*, $\:{n}_{p}^{2}$
Response to name	Response to name delay	0.077, 0.050	0.5, 0.009	0.88, 0	**< 0.05**,** 0.054**	0.64, 0.0031
	Proportion of name call responses	0.124, 0.027	0.25, 0.016	**< 0.05**,** 0.051**	0.055, 0.037	0.49, 0.0053
Gaze	Gaze percent social	0.204, 0.019	0.053, 0.043	**< 0.005**, 0.099*	**< 0.05**,** 0.052**	0.38, 0.0086
	Gaze speech correlation	**0.002**, 0.107*	0.74, 0.0013	**< 0.005**, 0.11*	**< 0.001**, 0.12*	0.68, 0.0019
	Gaze silhouette score	0.132, 0.026	**< 0.005**, 0.088*	**< 0.001**, 0.13*	0.47, 0.0053	0.7, 0.0016
Head movements	Rate of head movements during social movies	**< 0.001**, 0.158*	0.52, 0.0049	**< 0.001**, 0.14*	**< 0.05**,** 0.042**	0.2, 0.018
	Rate of head movements during nonsocial movies	0.146, 0.024	**< 0.01**,** 0.087** *	**< 0.001**, 0.15*	0.08, 0.031	0.3, 0.012
	Rate of head movements during social movies derivative	**< 0.001**, 0.138*	0.25, 0.016	**< 0.001**, 0.13*	**< 0.05**,** 0.047**	0.28, 0.013
	Rate of head movements during nonsocial movies derivative	0.165, 0.022	**< 0.01**,** 0.083** *	**< 0.001**, 0.15*	0.085, 0.03	0.27, 0.014
	Head movements complexity during social movies	**< 0.001**, 0.166*	0.47, 0.006	**< 0.001**, 0.18*	**< 0.005**, 0.091*	0.56, 0.0037
	Head movements complexity during nonsocial movies	**0.024**,** 0.058**	**< 0.05**,** 0.05**	**< 0.005**, 0.11*	**< 0.005**, 0.08*	0.22, 0.017
Facial expression	Eyebrow complexity during social movies	0.176, 0.021	0.39, 0.0085	0.65, 0.0026	**< 0.005**, 0.081*	0.11, 0.028
	Eyebrow complexity during nonsocial movies	0.267, 0.014	**< 0.05**,** 0.07** *	**< 0.01**, 0.082*	**< 0.05**, 0.06*	0.12, 0.027
	Mouth complexity during social movies	0.152, 0.024	0.16, 0.023	0.13, 0.029	**< 0.01**, 0.07*	0.079, 0.034
	Mouth complexity during nonsocial movies	0.248, 0.016	**< 0.005**,** 0.094** *	**< 0.005**, 0.1*	**< 0.05**,** 0.051**	0.12, 0.027
Facing forward	Social	**0.006**, 0.084*	0.31, 0.012	**< 0.005**, 0.12*	**< 0.005**, 0.098*	0.6, 0.003
	Nonsocial	0.330, 0.011	**< 0.01**,** 0.086** *	**< 0.005**, 0.13*	0.061, 0.035	0.14, 0.024
Blink	Social	0.201, 0.019	0.21, 0.019	**< 0.05**,** 0.057**	**< 0.05**, 0.062*	0.24, 0.015
	Nonsocial	0.929, 0	**< 0.05**,** 0.058**	**< 0.05**,** 0.07**	0.28, 0.012	0.24, 0.016
Touch	Pop Rate	**0.016**, 0.081*	0.11, 0.035	**< 0.005**, 0.12*	0.71, 0.0018	0.081, 0.045
	Exploratory percentage	0.126, 0.034	0.23, 0.02	**< 0.05**, 0.098*	0.47, 0.0069	0.17, 0.028
	Average applied force	**0.025**,** 0.072**	**< 0.01**,** 0.092** *	**< 0.001**, 0.17*	0.84, 0	0.079, 0.046
	Average length	0.068, 0.047	0.54, 0.0051	**< 0.05**,** 0.063**	0.33, 0.012	0.84, 0

Significant p-values in bold (uncorrected). * indicates results that survive FDR correction (q < 0.05) across the full family of contrasts. ANCOVA effect size: weak: $\:{n}_{p}^{2}$ ~0.01, medium: $\:{n}_{p}^{2}$ ~0.06, strong: $\:{n}_{p}^{2}$ ~0.14. Effect sizes below 0.001 shown as 0.

**Table 3:** Spearman correlations of SenseToKnow app features with Social Responsiveness Scale (SRS) subscales and ADHD Rating Scale (ADHD-RS) clinical measures in neurotypical (NT), ADHD, Autism, Autism+ADHD groups.

Digital phenotype	SenseToKnow feature	Social responsiveness scale						ADHD rating scale			
		Composite score		Social awareness		Restrictive and repetitive behaviors		Score			
		Autism	Autism+ ADHD	Autism	Autism+ ADHD	Autism	Autism+ ADHD	NT	ADHD	Autism	Autism+ ADHD
Response to name	Response to name delay	0.016	0.036	0.02	0.23	− 0.0096	0.29	0.25	0.035	− 0.044	− 0.086
	Proportion of name call responses	− 0.236	0.012	**− 0.29**	− 0.14	0.024	**0.34**	− 0.073	0.036	− 0.02	0.15
Gaze	Gaze percent social	0.049	0.031	**− 0.31**	− 0.40*	− 0.076	0.15	0.018	0.078	0.17	0.15
	Gaze speech correlation	− 0.22	**− 0.32**	− 0.62*	− 0.39*	− 0.48*	− 0.28	0.018	**− 0.37**	− 0.19	− 0.091
	Gaze silhouette score	0.033	0.093	− 0.22	− 0.056	− 0.18	− 0.029	**− 0.36**	0.09	− 0.036	0.17
Head movements	Rate of head movements during social movies	0.091	**0.37**	**0.27**	0.55*	0.22	0.065	0.22	0.18	0.27	− 0.067
	Rate of head movements during nonsocial movies	− 0.010	**0.35**	0.19	0.40*	0.25	0.17	0.12	0.23	0.21	0.087
	Rate of head movements during social movies derivative	0.071	**0.37**	0.32*	0.55*	0.22	0.088	0.26	0.19	0.25	− 0.089
	Rate of head movements during nonsocial movies derivative	− 0.095	30	0.12	**0.34**	0.19	0.11	0.068	0.21	0.16	0.062
	Head movements complexity during social movies	− 0.078	− 0.26	− 0.38*	− 0.28	**− 0.28**	− 0.054	− 0.20	− 0.23	− 0.10	0.072
	Head movements complexity during nonsocial movies	0.13	**− 0.32**	− 0.088	− 0.25	− 0.034	− 0.22	− 0.061	− 0.23	− 0.055	− 0.089
Facial expression	Eyebrow complexity during social movies	− 0.16	− 0.24	− 0.41*	− 0.095	− 0.21	**− 0.33**	0.079	− 0.051	0.052	− 0.14
	Eyebrow complexity during nonsocial movies	0.038	**− 0.40**	− 0.10	− 0.19	− 0.17	− 0.23	0.10	**− 0.29**	0.019	− 0.17
	Mouth complexity during social movies	− 0.15	− 0.22	− 0.38*	− 0.058	− 0.23	− 0.22	0.10	− 0.045	0.079	− 0.035
	Mouth complexity during nonsocial movies	− 0.025	− 0.26	− 0.13	− 0.037	− 0.2	− 0.25	0.0073	**− 0.29**	0.014	− 0.16
Facing forward	Social	− 0.22	**− 0.33**	− 0.38*	− 0.37*	− 0.37*	0.12	− 0.12	− 0.16	− 0.16	0.019
	Nonsocial	− 0.017	**− 0.31**	− 0.15	**− 0.34**	− 0.2	− 0.062	− 0.057	**− 0.32**	− 0.087	− 0.062
Blink	Social	0.25	0.25	0.49*	0.16	**0.27**	− 0.04	0.0098	0.04	**0.30**	0.21
	Nonsocial	0.21	0.18	0.39*	0.18	0.19	− 0.045	0.11	0.21	0.23	0.11
Touch	Pop Rate	0.0046	− 0.19	− 0.092	− 0.26	0.13	− 0.091	− 0.22	− 0.27	− 0.27	0.028
	Exploratory percentage	0.11	− 0.20	− 0.14	0.0094	− 0.0091	0.04	− 0.28	0.28	0.14	0.26
	Average applied force	− 0.087	− 0.029	− 0.10	− 0.0021	0.13	0.19	− 0.11	0.078	− 0.12	0.053
	Average length	− 0.068	− 0.17	0.044	− 0.23	− 0.11	0.24	0.12	− 0.15	0.16	− 0.029

Significant correlations (*p* < 0.05) shown in bold. *indicates correlations that survive FDR correction.

**Table 4:** Coefficients and significance of ordinary least squares regression for each SenseToKnow feature, with the feature as the dependent variable, and age along with indicator variables for if the subject has autism or has ADHD as independent variables. (*N* = 183).

Digital phenotype	SenseToKnow feature	Intercept	Autism	ADHD	Age
		Coef.	Coef.	Coef.	Coef.
Response to name	Response to name delay	0.878	-0.358	0.186	0.007
	Proportion of name call responses	0.346*	0.16** ^**†**^	0.023	0.001
Gaze	Gaze percent social	0.504***	-0.044*** ^**†**^	-0.005	0.0
	Gaze speech correlation	0.307***	-0.135*** ^**†**^	0.004	0.002*
	Gaze silhouette score	0.537***	-0.059*** ^**†**^	-0.033	0.0
Head movements	Rate of head movements during social movies	2.736***	1.018*** ^**†**^	0.336	-0.029***
	Rate of head movements during nonsocial movies	3.096***	1.166*** ^**†**^	0.158	-0.028*
	Rate of head movements during social movies derivative	0.018***	0.007*** ^**†**^	0.002	-0.0***
	Rate of head movements during nonsocial movies derivative	0.024***	0.01*** ^**†**^	0.001	-0.0*
	Head movements complexity during social movies	15.09***	-7.293*** ^**†**^	-1.035	0.119**
	Head movements complexity during nonsocial movies	5.907**	-3.981*** ^**†**^	-0.677	0.079**
Facial expression	Eyebrow complexity during social movies	24.007***	-2.366** ^**†**^	1.331	0.073*
	Eyebrow complexity during nonsocial movies	22.151***	-4.125*** ^**†**^	-0.094	0.067*
	Mouth complexity during social movies	22.761***	-2.703*** ^**†**^	0.425	0.084**
	Mouth complexity during nonsocial movies	22.101***	-3.987*** ^**†**^	-0.413	0.063
Facing forward	Social	82.707***	-9.589*** ^**†**^	0.175	0.143*
	Nonsocial	79.463***	-8.227*** ^**†**^	-1.085	0.148*
Blink rate	Social	0.002	0.003*** ^**†**^	-0.0	0.0
	Nonsocial	0.005*	0.002* ^**†**^	0.0	0.0
Touch	Pop Rate	0.785***	-0.033* ^**†**^	-0.039*	0.003***
	Exploratory percentage	96.549***	1.415	5.416	-0.406***
	Average applied force	0.427***	0.11* ^**†**^	0.114*	-0.005**
	Average length	11.241***	1.947* ^**†**^	0.233	-0.129***

*: *p* < 0.05, **: *p* < 0.01, ***: *p* < 0.001 (uncorrected). † indicates results that survive FDR correction (q < 0.05) across the 23 features within that predictor.

**Table 5:** Coefficients and significance of ordinary least squares (OLS) regression for each SenseToKnow feature, with the feature as the dependent variable, and age as a covariate (*N* = 183). Contains indicator variables for if the subject has autism or ADHD, with an additional interaction term for those with co-occurring autism and ADHD (autism+ADHD).

Digital phenotype	SenseToKnow feature	Intercept	Autism	ADHD	Autism +ADHD interaction effect	Age
		Coef.	Coef.	Coef.	Coef.	Coef.
Response to name	Response to name delay	0.715	-0.099	0.471	-0.478	0.007
	Proportion of name call responses	0.322*	0.196* ^**†**^	0.06	-0.074	0.001
Gaze	Gaze percent social	0.519***	-0.067*** ^**†**^	-0.030	0.049*	0
	Gaze speech correlation	0.315***	-0.148*** ^**†**^	-0.009	0.026	0.002*
	Gaze silhouette score	0.555***	-0.086*** ^**†**^	**-0.06**	0.055	0
Head movements	Rate of head movements during social movies	2.822***	0.886** ^**†**^	0.2	0.269	-0.029***
	Rate of head movements during nonsocial movies	2.702***	1.767*** ^**†**^	0.777	-1.231*	-0.027*
	Rate of head movements during social movies derivative	0.018***	0.007** ^**†**^	0.002	0.001	-0.0***
	Rate of head movements during nonsocial movies derivative	0.021**	0.015*** ^**†**^	0.007	-0.011*	-0.0*
	Head movements complexity during social movies	14.889***	-6.986*** ^**†**^	-0.718	-0.628	0.12**
	Head movements complexity during nonsocial movies	6.663***	-5.132*** ^**†**^	-1.864	2.358	0.077**
Facial expression	Eyebrow complexity during social movies	24.779***	-3.543** ^**†**^	0.117	2.411	0.072*
	Eyebrow complexity during nonsocial movies	23.803***	-6.643*** ^**†**^	-2.692	5.158**	0.064**
	Mouth complexity during social movies	23.826***	-4.326*** ^**†**^	-1.249	3.324*	0.082**
	Mouth complexity during nonsocial movies	23.708***	-6.436*** ^**†**^	-2.939*	5.016**	0.06
Facing forward	Social	84.181***	-11.836*** ^**†**^	-2.143	4.603	0.14*
	Nonsocial	83.029***	-13.662*** ^**†**^	-6.691*	11.132**	0.141*
Blink rate	Social	0.001	0.004*** ^**†**^	0.001	-0.002	0.0
	Nonsocial	0.003	0.004*** ^**†**^	0.002	-0.004*	0.0
Touch	Popping rate	0.779***	-0.024	-0.031	-0.017	0.003***
	Exploratory percentage	98.512***	-1.32	2.813	5.619	-0.414***
	Touch average applied force	0.428***	0.109	0.112	0.003	-0.005**
	Touch average length	10.856***	2.483*	0.744	-1.103	-0.128***

*: *p* < 0.05, **: *p* < 0.01, ***: *p* < 0.001 (uncorrected). † indicates results that survive FDR correction (q < 0.05) across the 23 features within that column.

### Clinical measures

Cognitive ability was assessed using the Differential Ability Scales, 2nd Edition (DAS-II^60^ prior to March 2020 and using the Wechsler Preschool and Primary Scale of Intelligence - Fourth Edition (WPPSI-IV^61^ or the Wechsler Intelligence Scale for Children-Fifth Edition (WISC-V^62^ afterwards, depending on the child’s age. A developmental quotient (DQ) was computed to have an age-standardized measurement across instruments.

Adaptive functioning was measured using the VABS-3, based on a standardized caregiver report, with subscales based on adaptive, motor, daily living, communication, and socialization skills. Autism-related social communication and repetitive behaviors were assessed using the SRS-2. Analyses used the SRS-2 total score alongside the social awareness (SA) and restrictive and repetitive behavior (RRB) subscales.

### SenseToKnow app computer vision and tactile features

The SenseToKnow app was administered in a laboratory setting under the supervision of a trained research assistant. Children viewed a 10-minute series of short movies followed by playing a bubble popping touchscreen game on an iPad mounted in front of them (see Perochon et al.^27^ for additional details). Movies were short, dynamic, and strategically designed to elicit autism-relevant behaviors and included both social movies, such as actors talking to one another, and nonsocial movies, such as floating bubbles or toys. Movies featured actors from diverse ethnic and racial backgrounds and were available in both English and Spanish. While videos were available in Spanish, all participants in the present study’s cohort viewed English versions. Spinning Top, Make Me Laugh, and Rhymes movies were updated to be developmentally appropriate for this older cohort; all other movies were identical to previous administrations of SenseToKnow in toddlers (Fig. 1). Additionally, previous administrations of SenseToKnow in toddlers^27^ often had the child perform the administration while in their parent’s lap; in this older cohort they sat in their own chair.

Videos were recorded using the iPad’s front-facing camera at 30 frames per second in 1920 × 1080 resolution. Faces were automatically detected using RetinaFace^63^ and manually verified. An automated CVA validity algorithm was applied to ensure only high-quality administrations were used, rating videos based on the child’s position and orientation towards the screen^28^. During the bubble popping game, the device’s touchscreen sensor data was recorded.

From video and touchscreen data, a series of previously validated computer vision and inertial features were computed, related to response to name, gaze, head movements, facial movement patterns, attention, and popping game performance (Fig. 1). Features were computed separately during social and nonsocial movies where applicable.

Response to name was assessed at three predetermined points, recording the proportion of name calls the participants responded to as well as their delay in response^29^. The participant’s estimated gaze location was extracted using a deep neural network^64^ using the child’s head pose and eye landmarks. From this, the proportion of gaze at social stimuli (gaze percent social), the correlation between the child’s alternating gaze and two actors’ speech (gaze speech correlation), and a metric of how well the child’s gaze patterns cluster/focus cohesively (gaze silhouette score) were computed^30^. Head movement features were based on the rate and acceleration of facial landmarks^31,32^. The complexity of head and facial movement features was computed using multiscale entropy^65^, where higher complexity values indicated movement patterns that were less predictable^33^. Proxies for attention were computed through blink rate, using facial action units, and how often the child was facing forward towards the screen, using the roll, pitch, and yaw angles of the child’s head. Lower blink rate and higher proportion facing forward are used as a proxy for increased attention towards the stimuli being shown^34^. Inertial features from the bubble popping game included the ratio of touches that lead to popped bubbles, the percentage of the screen the child explored, and the average applied force length of touch events^35^. Each of these features was validated in independent samples and the computation process for each feature was identical to the prior studies that introduced these features.

### Statistical analyses

#### Group differences

We used analysis of covariance with age as a covariate for all group comparisons of SenseToKnow features. Although many features were non-normally distributed (Shapiro-Wilk), analysis of covariance is robust to non-normality at these group sizes (*n* = 41–53)^66^. For each feature we report the age-adjusted group difference. To account for multiple comparisons, Benjamini-Hochberg false-discovery-rate correction was applied across the full family of comparisons; results are reported as significant only when they survive this correction.

We performed an exploratory analysis of associations between SenseToKnow features and clinical measures within each diagnostic group, computed using Spearman’s correlation. This within-group approach avoids conflating within-group associations with between-group differences. Correlations were computed between app features and SRS-2 total and subscale scores (SA and RRB), VABS-3 subdomain scores, and ADHD-RS scores. For the feature and symptom severity correlations (SRS-2 and ADHD-RS), Benjamini-Hochberg FDR correction was applied within each measure-by-group family. Whilst these analyses were exploratory, this correction allowed for better interpretation of the significance of these results.

#### Regression analysis

To assess both the independent and joint effects of autism and ADHD diagnostic status on app feature outcome, we fit ordinary least squares (OLS) regression models for each feature with binary indicators for autism (*hasAutism*) and ADHD (*hasADHD*), and their interaction term (*hasAutism*$\:\times\:$*hasADHD*), with age as a covariate:

$$\:feature=\:{\beta\:}_{0}+\:{\beta\:}_{1}\times\:hasAutism+\:{\beta\:}_{2}\times\:hasADHD+ {\beta\:}_{3}\times\:(hasAutism\times\:hasADHD)+{\beta\:}_{4}\times\:age.$$

To directly compare the effect of autism or ADHD status on app feature outcome, linear contrast tests were used to test whether coefficients differed in magnitude ($\:{\beta\:}_{1}={\beta\:}_{2}$). For contrast tests, analysis was restricted to the autism and ADHD groups with the neurotypical participants as a baseline. Afterwards, to understand the impact and significance of having autism+ADHD and how this may differ from the autism and ADHD groups, we analyzed the interaction effect across all groups. Interaction effects were interpreted based on the sign of the interaction term coefficient $\:{(\beta\:}_{3})$. If found to be statistically significant, this term shows whether the autism+ADHD group has a positive interaction where the autism+ADHD effects are higher than the additive sum of separate autism and ADHD coefficients ($\:{\beta\:}_{3}>0$), no interaction where the autism+ADHD effects are equivalent to the sum of separate autism and ADHD coefficients ($\:{\beta\:}_{3}=0$), or a negative interaction where the autism+ADHD effects are smaller than the sum of separate autism and ADHD coefficients ($\:{\beta\:}_{3}<0$), when compared to the effect of an autism or ADHD diagnosis on feature outcome. The OLS model framework was chosen for its clear interpretability, and results were taken in context alongside group comparisons from the previous analysis. To account for multiple comparisons, Benjamini-Hochberg false-discovery-rate correction was applied within each predictor.

### SenseToKnow feature reliability

A subset of children completed the SenseToKnow app on more than one occasion (94 children: 10 neurotypical, 27 with ADHD, 34 with autism, and 23 with co-occurring autism and ADHD). Because test-retest reliability reflects the measurement stability of each feature rather than a characteristic of a diagnostic group, and because the number of repeat administrations within each group was small (particularly for neurotypical children), we estimated reliability across the pooled repeat sample rather than within each group. Reliability of each feature was estimated using the intraclass correlation coefficient (two-way random effects, absolute agreement, single measurement; ICC(A,1)), with 95% confidence intervals bootstrapped across children.

### Symptom measure overlap

The overlap between the SRS-2 and the ADHD Rating Scale was quantified by their Pearson correlation across the sample and by a partial correlation adjusting for diagnostic group, age, and developmental quotient.

## Supplementary Information

Below is the link to the electronic supplementary material.

Supplementary Material 1

## Data Availability

Individual-level descriptive information from this study is archived and made accessible to the research community through the National Institute of Mental Health National Data Archive (NDA), in accordance with provisions defined in the Duke University Institutional Review Board and the NDA Data Sharing Policy. Please contact the corresponding author to inquire about starting this process.

## Code availability

The analysis code supporting the findings of this study is openly available in Zenodo at https://doi.org/10.5281/zenodo.21479562. The repository contains the scripts that produce the age-adjusted group-difference, regression, within-group correlation, test-retest reliability, and symptom-scale overlap analyses reported here, together with documentation of the required input-data format. The SenseToKnow computer-vision and touchscreen features consumed by this code were derived using previously published, independently validated methods described in the Methods.
