# Supplementary material for: Digital phenotyping captures autism-associated behaviors in preschool- and school-age autistic children with and without co-occurring ADHD
Source: Sci Rep. 2026 Aug 12;16:37643. doi: 10.1038/s41598-026-65395-7 (PMC13751302; doi:10.1038/s41598-026-65395-7)
Supplement: Supplementary file 1 [file 41598_2026_65395_MOESM1_ESM.docx]

**Supplementary Material**

**Table S1.**

**Correlation with age for each SenseToKnow feature in neurotypical (NT), ADHD, Autism, and co-occurring autism and ADHD (Autism+ADHD) groups.**

| Digital Phenotype | SenseToKnow Feature | NT | | ADHD | | Autism | | Autism+ADHD | |
| --- | --- | --- | --- | --- | --- | --- | --- | --- | --- |
|  |  | Corr. | p | Corr. | p | Corr. | p | Corr. | p |
| Response to  Name | Response to name delay | 0.42 | 0.06 | 0.23 | 0.19 | 0.08 | 0.63 | -0.18 | 0.34 |
|  | Proportion of name call responses | -0.11 | 0.48 | -0.16 | 0.28 | 0.26 | 0.06 | 0.07 | 0.68 |
| Gaze | Gaze at social stimuli | 0.04 | 0.79 | -0.08 | 0.59 | 0.03 | 0.85 | 0.18 | 0.26 |
|  | Gaze speech correlation | 0.29 | 0.06 | 0.13 | 0.39 | 0.14 | 0.33 | 0.12 | 0.44 |
|  | Gaze silhouette score | -0.16 | 0.32 | 0.16 | 0.27 | 0.2 | 0.16 | -0.01 | 0.96 |
| Head  Movements | Rate of head movements during social movies | **-0.37** | **<.05** | -0.22 | 0.13 | **-0.35** | **<.05** | -0.08 | 0.63 |
|  | Rate of head movements during nonsocial movies | **-0.34** | **<.05** | -0.27 | 0.07 | -0.15 | 0.3 | -0.06 | 0.72 |
|  | Rate of head movements during social movies derivative | **-0.42** | **<.01** | **-0.28** | **<.05** | **-0.29** | **<.05** | -0.11 | 0.48 |
|  | Rate of head movements during nonsocial movies derivative | -0.27 | 0.09 | -0.25 | 0.08 | -0.16 | 0.26 | -0.01 | 0.95 |
|  | Head movements complexity during social movies | 0.19 | 0.24 | 0.17 | 0.24 | **0.35** | **<.05** | -0.11 | 0.51 |
|  | Head movements complexity during nonsocial movies | **0.33** | **<.05** | **0.32** | **<.05** | 0.19 | 0.17 | -0.01 | 0.97 |
| Facial Expression | Eyebrow complexity during social movies | 0.26 | 0.1 | -0.06 | 0.67 | **0.4** | **<.01** | 0 | 0.99 |
|  | Eyebrow complexity during nonsocial movies | 0.21 | 0.2 | 0.19 | 0.19 | 0.07 | 0.62 | 0.05 | 0.73 |
|  | Mouth complexity during social movies | 0.28 | 0.08 | 0.28 | 0.05 | **0.33** | **<.05** | 0.05 | 0.76 |
|  | Mouth complexity during nonsocial movies | 0.21 | 0.19 | **0.3** | **<.05** | 0.07 | 0.62 | 0.09 | 0.57 |
| Facing  Forward | Time spent facing toward screen during social movies | 0.06 | 0.7 | 0.16 | 0.28 | 0.23 | 0.09 | -0.04 | 0.8 |
|  | Time spent facing toward screen during nonsocial movies | 0.1 | 0.53 | **0.31** | **<.05** | 0.12 | 0.38 | -0.01 | 0.97 |
| Blink | Blink rate during social movies | 0.16 | 0.36 | 0.11 | 0.47 | 0.03 | 0.84 | 0.14 | 0.41 |
|  | Blink rate during nonsocial movies | 0.04 | 0.82 | -0.02 | 0.87 | -0.02 | 0.9 | 0.14 | 0.38 |
| Touch | Touch popping ratio | 0.09 | 0.59 | **0.55** | **<.001** | **0.32** | **<.05** | **0.37** | **<.05** |
|  | Touchscreen exploratory percentage | **-0.52** | **<.01** | **-0.51** | **<.001** | -0.06 | 0.72 | **-0.6** | **<.001** |
|  | Touch average applied force | 0.06 | 0.73 | -0.11 | 0.48 | 0 | 0.99 | -0.23 | 0.22 |
|  | Touch average length | **-0.74** | **<.0001** | **-0.49** | **<.01** | -0.25 | 0.13 | **-0.42** | **<.05** |

**Table S2. Test-retest reliability of SenseToKnow features (n = 94 repeat administrations, pooled across diagnostic groups).**

| Digital Phenotype | SenseToKnow Feature | N | ICC(A,1) | 95% CI |
| --- | --- | --- | --- | --- |
| Response to Name | Response to name delay | 62 | 0.28 | 0.01–0.50 |
|  | Proportion of name call responses | 94 | 0.58 | 0.42–0.71 |
| Gaze | Gaze at social stimuli | 94 | 0.43 | 0.26–0.56 |
|  | Gaze speech correlation | 94 | 0.63 | 0.47–0.76 |
|  | Gaze silhouette score | 94 | 0.50 | 0.33–0.66 |
| Head Movements | Rate of head movements during social movies | 94 | 0.67 | 0.53–0.78 |
|  | Rate of head movements during nonsocial movies | 94 | 0.57 | 0.33–0.72 |
|  | Rate of head movements during social movies derivative | 94 | 0.63 | 0.47–0.77 |
|  | Rate of head movements during nonsocial movies derivative | 94 | 0.49 | 0.19–0.80 |
|  | Head movements complexity during social movies | 94 | 0.78 | 0.70–0.86 |
|  | Head movements complexity during nonsocial movies | 94 | 0.76 | 0.66–0.83 |
| Facial Expression | Eyebrow complexity during social movies | 94 | 0.49 | 0.29–0.64 |
|  | Eyebrow complexity during nonsocial movies | 94 | 0.53 | 0.35–0.68 |
|  | Mouth complexity during social movies | 94 | 0.53 | 0.35–0.67 |
|  | Mouth complexity during nonsocial movies | 94 | 0.52 | 0.29–0.69 |
| Facing Forward | Time spent facing toward screen during social movies | 94 | 0.74 | 0.61–0.84 |
|  | Time spent facing toward screen during nonsocial movies | 94 | 0.71 | 0.56–0.82 |
| Blink | Blink rate during social movies | 94 | 0.67 | 0.49–0.79 |
|  | Blink rate during nonsocial movies | 94 | 0.50 | 0.39–0.69 |
| Touch | Touch popping ratio | 72 | 0.69 | 0.48–0.82 |
|  | Touchscreen exploratory percentage | 72 | 0.61 | 0.45–0.74 |
|  | Touch average applied force | 70 | 0.25 | 0.00–0.47 |
|  | Touch average length | 72 | 0.27 | 0.18–0.62 |

**Figure S1. Age distribution by diagnosis.**
